# The Psychological Challenges of Replacing Conventional Karyotyping with Genomic SNP Array Analysis in Prenatal Testing

**DOI:** 10.3390/jcm3030713

**Published:** 2014-07-03

**Authors:** Sam Riedijk, Karin E. M. Diderich, Sanne L. van der Steen, Lutgarde C. P. Govaerts, Marieke Joosten, Maarten F. C. M. Knapen, Femke A. T. de Vries, Diane van Opstal, Aad Tibben, Robert-Jan H. Galjaard

**Affiliations:** 1 Department of Clinical Genetics, Erasmus Medical Centre, P.O. Box 2040, Rotterdam 3000 CA, The Netherlands; E-Mails: k.diderich@erasmusmc.nl (K.E.M.D.); s.vandersteen@erasmusmc.nl (S.L.S.); l.c.p.govaerts@erasmusmc.nl (L.C.P.G.); a.joosten@erasmusmc.nl (M.J.); f.a.t.devries@erasmusmc.nl (F.A.T.V.); a.vanopstal@erasmusmc.nl (D.O.); a.tibben@lumc.nl (A.T.); r.galjaard@erasmusmc.nl (R.-J.H.G.); 2 Department of Obstetrics and Prenatal Medicine, Erasmus Medical Centre, 3015 CN Rotterdam, The Netherlands; E-Mail: m.knapen@erasmusmc.nl; 3 Stichting Prenatale Screening Zuidwest Nederland, 3015 CN, Rotterdam, The Netherlands; 4 Department Clinical Genetics, Leiden University Medical Centre, 2300 RC Leiden, The Netherlands

**Keywords:** prenatal diagnosis, SNP array analysis, counselling challenges

## Abstract

Pregnant couples tend to prefer a maximum of information about the health of their fetus. Therefore, we implemented whole genome microarray instead of conventional karyotyping (CK) for all indications for prenatal diagnosis (PND). The array detects more clinically relevant anomalies, including early onset disorders, not related to the indication and more genetic anomalies of yet unquantifiable risk, so-called susceptibility loci (SL) for mainly neurodevelopmental disorders. This manuscript highlights the psychological challenges in prenatal genetic counselling when using the array and provides counselling suggestions. First, we suggest that pre-test decision counselling should emphasize deliberation about what pregnant couples wish to learn about the future health of their fetus more than information about possible outcomes. Second, pregnant couples need support in dealing with SL. Therefore, in order to consider the SL in a proportionate perspective, the presence of phenotypes associated with SL in the family, the incidence of a particular SL in control populations and in postnatally ascertained patients needs highlighting during post-test genetic counselling. Finally, the decision that couples need to make about the course of their pregnancy is more complicated when the expected phenotype is variable and not quantifiable. Therefore, during post-test psychological counseling, couples should concretize the options of continuing and ending their pregnancy; all underlying feelings and thoughts should be made explicit, as well as the couple’s resources, in order to attain adequate decision-making. As such, pre- and post-test counselling aids pregnant couples in handling the uncertainties that may accompany offering a broader scope of genetic PND using the array.

## 1. Background

Until recently, conventional karyotyping (CK) was the gold standard to detect chromosomal aberrations in prenatal diagnosis (PND). Whole genome microarray analysis has been introduced in prenatal medicine due to its evident advantages; microarray detects more submicroscopic copy number variants (CNVs) that cause clinically relevant abnormalities [1,2,3] and generates results faster than CK. 

It is remarkable that ample publications voice healthcare professionals’ concerns regarding high resolution invasive prenatal genetic diagnosis [4,5,6], but few studies have inspected what pregnant couples want to learn about the future health of their fetus [7]. Since we started microarray analysis (in our case, SNP array) in the case of fetal anomalies in 2009, we offered pregnant couples a choice regarding the extent to which they wished to be informed about predefined categories of genetic anomalies [8]. The majority of pregnant couples chose to be informed of any (susceptibility for) severe condition(s) of any age of onset. Another study reported that a majority of pregnant women with advanced maternal age (AMA) and/or an increased risk for a fetus with Down, Edwards and Patau syndrome based on first trimester screening (FTS) preferred diagnostics of all chromosome anomalies by CK over targeted genetic diagnostics for trisomy 13, 18 and 21 (by Quantitative fluorescence PCR or Multiplex Ligation-dependent Probe Amplification) [9]. Yet, another study demonstrated that only a slight majority of pregnant couples preferred CK over targeted genetic diagnostics [10]. A recent study involving focus groups offering a hypothetical choice revealed that individualized choice was highly valued [11]. The literature indicates a preference among a group of pregnant couples to learn as much as possible from PND. Moreover, those pregnant couples indicated that they wish to make their own decisions regarding the scope of PND. It therefore seems to be justified to offer pregnant couples the SNP array for all indications in PND. 

Our lab in Rotterdam has completely replaced CK by a whole genome SNP array for all indications since July, 2012 [12,13]. Thus, prenatal genetic testing for increased risk of prenatal common occurring aneuploidies, like Down syndrome, is also performed by the SNP array. However, for these indications compared to the indication of ultrasound anomalies, the analysis of the genetic variants is lowered from 0.15 to 0.5 Mb to reduce, e.g., the number of genetic variants of unknown clinical significance [13]. We only communicate pathogenic array findings, which means that variants of unknown clinical significance (VOUS) are not reported. We recognize three types of pathogenic aberrations in the fetus: causative findings (explaining the phenotype), unexpected diagnoses, early onset disorders not being related to the indication, for instance a case of Duchenne muscular dystrophy, and susceptibility loci (SL) for mainly neurodevelopmental disorders [14,15].

Whereas PND has always been familiar with the phenomenon of unexpected diagnoses, the number and nature of unexpected diagnoses has expanded with the implementation of the SNP array when compared to CK [16,17]. We recently detected a CNV causing Duchenne muscular dystrophy in a patient at increased risk for Down syndrome based on first trimester screening. Such a condition would not have been detected by CK. These are the types of cases in which we and our patients perceive the SNP array to be of additional value over CK. We anticipated detecting more severe untreatable late onset disorders, as well. We agreed that if a genetic predisposition to a severe, untreatable late onset condition was detected, we would determine how to deal with this finding in a multidisciplinary team. We acknowledge the difficulties of both safeguarding the child’s right not to know and the reproductive interest of the parents [5], and we approach this matter with a case-by-case policy. To date, we have not detected any late onset disorder using the SNP array in over 2000 cases [18,19].

The most challenging type of SNP array results are the SL. SL are pathogenic CNVs (therefore, by definition, not VOUS), with incomplete penetrance or variable expression, that are associated with a yet unquantifiable chance for (mainly) neurodevelopmental disease (developmental delay, behavioral problems, learning problems, autism spectrum disorders and/or seizures) [14,20]. They are enriched in patient populations, but also present in normal controls. The mechanism underlying the pathogenicity is unknown and may be influenced by another genomic alteration [21]. Besides a heritable component, environmental factors may also influence the pathology of neurodevelopmental disease. An example of a CNV, which we frequently encounter, is a 0.5-Mb deletion at 15q11.2. Postnatal ascertainment was reported by Murthy *et al.* (2013) [22] and Doornbos *et al.* (2009) [23]. 

One recent study suggests that the information of SL may represent a burden to couples and that offering such knowledge should be accompanied by careful pre- and post-test counselling [24]. Concerns have been raised about the possible emotional harm indeterminate findings may cause to pregnant couples [25]. Another study stated that uncertain test results do not enable choice, and therefore, the burden of the anxiety and stress of uncertain test results is not justified. Some have argued that the burden of generating SL likely outweighs the possible benefits of detecting more pathogenic findings [1,13]. Others argue that it is paternalistic to withhold any outcomes from PND [25]. Instead of withholding outcomes, it has been suggested that an improved approach to reduce parental uncertainty is needed in counselling [26,27,28].

Our rationale to decide in favor of the disclosure of SL lies in the necessity of the follow-up of a pregnancy postnatally in order to learn to interpret the SL. If we do not disclose, we cannot justify follow-up. In addition, if we do not disclose and neurodevelopmental problems present in the child, we will have deprived the parents of important information, which might have led to more adequate diagnostics and care. On the other hand, we are well aware of the burden SL might present psychologically. Here, we provide an overview of the possible psychological and counselling challenges, and we share our ideas regarding how to deal with these.

Due to the challenges of detecting unexpected diagnoses and SL at relatively low prevalence, there is currently no consensus regarding the introduction of the array for all indications in prenatal medicine. With this paper, we aim to contribute to the development of international consensus. 

## 2. Pre-Test Challenges of Replacing CK with SNP Array in PND

### 2.1. Informed Decision-Making and Consent

Informed consent is a condition *sine qua non*; before decision-making, pregnant couples need to clearly oversee the possible consequences of engaging in prenatal genetic diagnosis. Informed decision-making has been defined as the outcome of a process based on sufficient knowledge and deliberation and that is congruent with one’s personal attitudes [29]. Adequate knowledge had a beneficial effect on psychological management of prenatal test decisions [30]. Conversely, uninformed decision-making had adverse effects on pregnant couples. Pregnant women who were unprepared for unfavorable screening results seemed to have more difficulty coping and with decision-making [31]. Informed decision-making was already a challenge before the introduction of the SNP array. Most pregnant women did not make informed decisions with respect to participation in prenatal screening [31,32], and baseline knowledge and reflection in women participating in prenatal screening was largely insufficient to prepare them for possible adverse outcomes [33]. With the SNP array, it has become even more difficult to obtain true informed consent. It is impossible both to provide (for the professional) and to process (for the couple) information on the level of specific diseases and related outcomes when the test may identify a multitude of diseases or (unquantifiable) health risks [5]. It is the question whether the alternative for informed consent, generic consent, provides sufficient basis for informed decision-making when pregnant couples have to anticipate the possibility of receiving results, like SL [5,34]. One could argue that categories of outcomes are not sufficiently specific for pregnant couples to fully grasp the consequences of receiving an unfavorable test result. In addition, generic categories may also be difficult to understand. To our knowledge, at present, data on the efficacy of generic categories are not available. Since understanding and overseeing the possible outcomes become increasingly difficult using the SNP array, we recommend that more emphasis should be placed on the dialogue during pre-test counselling regarding what pregnant couples wish to learn about the future health of their fetus.

### 2.2. Pre-Test Decision Counselling and Dialogue

Often underestimated aspects of counselling are the facilitation of understanding the information, attributing personal meaning to the information and decision-making based upon the information provided [35,36]. However, these aspects are important, because research has shown that if decisions made regarding PND are not fully congruent with feelings and personal, moral values and attitudes, the adaptation to the decision may be jeopardized, and the quality of life and partner-relationship may be adversely affected [37]. In fact, a truly informed decision is the outcome of a process of weighing and considering what the prospective parents consider to be a worthy life for their child, what termination of pregnancy would mean to them and whether some conditions would justify this decision. We recommend that such facilitation is accomplished by inviting pregnant couples to consider what they wish to know about the (future) health of the fetus and whether they wish to engage in PND. The ensuing dialogue between the pregnant couple and the healthcare provider will clarify, both to the pregnant couples and the professional, what conditions pregnant couples consider acceptable or not, which conditions would incline them to terminate the pregnancy and which conditions they would wish to prepare themselves for mentally. The outcomes of the deliberation may guide which technique for PND to use (targeted aneuploidy detection or SNP array analysis for the indication of AMA, for instance). Moreover, pregnant couples will become more aware of the possibilities and impossibilities of PND when they have been enabled to verbalize how PND results might affect them. Furthermore, pregnant couples will be more aware of their motivation for PND and how SL might affect them, thus preparing them for uncertainties. 

In summary, we promote that in addition to the provision of information, during pre-test counselling, more attention is paid to enabling pregnant couples to consider what they wish to learn from prenatal diagnosis. Couples need to be invited to personalize the information by reflecting on what possible outcomes might be generated and what consequences they would attach to various outcomes (*i.e.*, Down syndrome, late onset disorders, SL).

As such, pre-test counselling is the most important tool at hand for supporting pregnant couples in dealing with the possibility of receiving prenatal test results that are expected, unexpected or of unquantifiable risk.

## 3. Post-Test Challenges of Replacing CK with the SNP Array in Prenatal Genetic Testing

Research has shown that, irrespective of the decision, bearing the responsibility for the continuation or termination of pregnancy (TOP) might be traumatizing [35,38,39]. An extensive longitudinal study on the impact of late TOP found that as much as 46% of women were still traumatized 16 months after their termination of pregnancy [37]. Doubts in the decision period and prognostic uncertainty were associated with vulnerability to complicated grief and trauma up to four years after TOP [39,40]. Now that more disorders can be tested, PND may thus consider that more couples will experience doubts during the decision period and will have more difficulty in psychologically adjusting to a TOP. Post-test decision counseling is important, because it was proven to favorably influence feelings of doubt and uncertainty during the decision period [35]. What is new about posttest decision counseling in PND using the SNP array is how to aid pregnant couples in dealing with SL. 

### 3.1. Post-Test Genetic Counselling

It has been consistently reported that understanding and recall of information about (genetic) probabilities and chances are poor [41,42]. When outcomes are uncertain, like is the case with SL, these may be even more challenging to understand [15]. Uncertain outcomes have been shown to induce stress and impede decision-making [43]. The appraisal of the severity of possible outcomes may become more influential on the decision when outcomes are uncertain [44]. Understandably, the first reaction of some pregnant couples may be to panic about outcomes of unquantifiable expression. Taking the time to recover a sense of trust in the possibility of a favorable outcome and in one’s ability to cope with uncertainty is an essential task of posttest counselling. 

This psychological task is more difficult with the increase of unquantifiable results, and more couples will be confronted with this when using the SNP array in PND*.* Several sessions of posttest genetic counselling may be needed and should be available for the pregnant couple to learn to understand and provide personal meaning to an anomalous test outcome.

Since we started to employ the array for all PND indications, we have decided to discuss whether or not to disclose the SL to the patient in a multidisciplinary team. If disclosed, we currently offer these pregnant couples posttest genetic counseling by a clinical geneticist and by a psychologist, if needed. The clinical geneticist informs the pregnant couples about the incidence of this particular SL in control populations and in postnatally ascertained patient populations. The difference between postnatal ascertainment (the array was performed because of an existing health problem) and the prenatal identification of an SL, because of AMA, for instance, is explained. This approach puts the SL in a proportionate perspective. Furthermore, the specific abnormal phenotypes found after postnatal ascertainment are discussed. It is specified that the chance of an abnormal phenotype after prenatal diagnosis is currently not known in spite of efforts to quantify this [20], since it will depend on yet unknown other factors. We offer to analyze the prospective parents’ DNA with targeted SNP array as well, as it may be reassuring that a healthy parent is also carrying the SL. We do not propose to study all parents before counseling, because the detection rate of SL is relatively low and the genetic analysis is too laborious and is too costly for the parents. If abnormalities visible on a prenatal ultrasound, e.g., a congenital heart anomaly, have been described as being associated with this SL, we offer a detailed ultrasound follow-up examination during pregnancy, as well. 

Thus, we aim to put an SL in a realistic perspective for pregnant couples. The SL implies an increased risk of an abnormal phenotype. We consider SL as actionable findings, leading to extra attention postnatally for development and behavior and easy access to early intervention programs, if necessary.

### 3.2. Post-Test Psychological Counselling

In general, psychological counseling after the disclosure of unfavorable results from PND implies guidance and support in decision-making and the grief process [45]. It may be expected that in the case of outcomes of variable and uncertain phenotypes, the psychological process will become even more complex. 

Couples deciding about ending or continuing their pregnancy often do not have a clear view on what they might expect from either option. When a couple decides to continue their pregnancy, the counseling focuses on adjustment until and after delivery [45,46]. When a couple decides to terminate the pregnancy, the counselor focuses on preparation of the termination and adjustment thereafter. Therefore, we recommend that posttest decision counselling focuses on concretizing both options as specifically as possible, using written information, case scenarios and visualization techniques. Findings of unquantifiable expression of a certain phenotype, generated by the array, may be difficult if not impossible to concretize in scenarios. If a couple considers TOP because of an SL, it is important that the underlying feelings and thoughts are made explicit. Thus, underlying anxiety may either be reassured or made explicit as valid arguments for consideration in the decision. Pregnant couples may feel their resources to be insufficient for dealing with an adverse outcome. A lack of trust in their resources (*i.e.*, social, financial, emotional resources, *etc.*) may incline the couple to prefer to prevent the fetus’ possible suffering. Conversely, couples may accept the risk, because they rely on the possibility of a favorable outcome or on their resources for dealing with unfavorable outcomes. Awareness of such underlying feelings and thoughts may aid pregnant couples in making a decision that is complete and does justice to their capabilities and resources. This may be even more important in the case of SL, because pregnant couples can hardly anticipate the possible consequences. This approach is important to prevent couples from panicking into a decision and to ensure that the decision made is one that has taken into account all of the possible considerations (practical, medical, ethical, emotional, *etc.*) of the pregnant couple. 

Because of the difficulties SL may represent to pregnant couples, we are currently conducting a study in order to evaluate how pregnant couples regard SL, whether they wish to be informed of these and how they respond when confronted with SL. 

## 4. Summary and Conclusions

In summary, the array increases tested disorders in genetic PND. This broadening of the scope of PND may be accompanied by a number of psychological challenges for pregnant couples: Deciding what they wish to learn about the (future) health of their fetus, understanding (categories of) the possible outcomes of PND, dealing with unexpected diagnoses and SL, deciding about the course of their pregnancy and adjusting to the decision outcome. Unexpected prenatal test results and SL may complicate deliberation and decision-making regarding the course of pregnancy. During pre-test counselling, we suggest that the counsellor not only provides information regarding possible test outcomes, but also enables deliberation about what pregnant couples wish to learn about the health of their fetus. In addition, it must be specified that array results may not be as certain as pregnant couples might hope for. Increasing the number of genetic disorders in one test in PND will generate more results of variable expressivity and heterogeneity of clinical features, which may induce stress and impede decision-making [43]. We therefore recommend that several posttest genetic counseling sessions should be available for the pregnant couple in order to reach understanding and provide personal meaning to the test result. This is necessary to prevent couples from panicking into a decision. In the case of SL, we recommend that the clinical geneticist informs the pregnant couples about the incidence of this particular SL in control populations and in postnatally ascertained patient populations. This approach enables couples to consider the SL in a proportionate perspective.

With regard to posttest psychological counseling, we recommend that couples are encouraged to concretize as much as possible the options of continuing and ending their pregnancy. All underlying feelings and thoughts should be made explicit, as well as the couple’s resources for adequate decision-making. 

Pretest genetic and decision counselling and posttest genetic and psychological counselling should be readily available when offering a broader scope of genetic PND using the array [47]. This manuscript has provided insight into the specific psychological challenges that should be addressed and shared our ideas on how these may be handled during this counselling. Our suggestions will hopefully contribute to reaching international consensus on how to deal with the challenging outcomes of the array in PND and will provide ideas regarding how pregnant couples may best be supported. 
